# Eleventh Annual Meeting of the American Medical Association

**Published:** 1858-07

**Authors:** 


### ELEVENTH ANNUAL MEETING OF THE AMERICAN MEDICAL ASSOCIATION.

The following remarks were intended to accompany the report of proceedings in the June number of the Journal, but were crowded out by a surplus of matter.

In the June number of the Journal we gave a pretty full report of the proceedings of the recent annual meeting of the American Medical Association, held in Washington, D. C., compiled from the reports in the *Daily Washington Union.* The number of delegates and permanent members in attendance was large; and, *socially*, it was one of the most pleasant meetings since the organization of the association. The very generous hospitality of the local profession in the cities of Washington and Georgetown, aided by the brilliant receptions at the President's house and the residence of Senator Douglas, together with the trip on the Potomac river to Mount Vernon, combined to render the meeting one that will ever be remembered with pleasure, by those who were so fortunate as to enjoy it. We wish we could speak in the same unqualified terms of commendation concerning the business transacted, and the manner of doing it.

But a due regard for truth compels us to say, that while *socially* the meeting in Washington was one of the most pleasant we ever attended, in a business and scientific aspect it was decidedly the most barren and unprofitable. From a total ignorance of parliamentary rules on the part of the highly-esteemed and worthy presiding officer, much of the business was done in a manner so irregular and hesitating, as to mar its interest; and so much time was consumed in enforcing the ethical rules brought to bear upon the conduct of Drs. Reese of N. Y., and Bryan of Philadelphia, in relation to James

McClintock of the latter city; and in a strenuous effort to induce the association to interfere directly in the appointment of drug inspector for the city of New York,—that very little time was left for the presentation of scientific papers or the reports of committees.

The only reports read were one by Dr. J. R. Wood, from the special committee on a definite plan of instruction to be given in the medical colleges, and one by Dr. A. B. Palmer, from the standing committee on medical literature. The first contained little else than the same general propositions which were made and approved at the first organization of the association in 1847; while the latter possessed in a much greater degree the qualities of a literary essay in the *hyfalutin* style, than of a plain, candid report on the medical literature of our country. The reports from special committees were not called until the last day of the session; and when about two-thirds of those on the list had been called and disposed of, a motion was made and adopted, that the remaining reports from special committees be called, read by their titles, and referred to the committee of publication.

After the adoption of this motion, the association proceeded immediately to other business, thus leaving several committees without an opportunity of even stating whether they had prepared reports or not. And in the official record of proceedings as published in the *Medical Reporter*, over the signatures of the secretaries, both the motion itself and the committees cut off by it, are entirely omitted. And yet, during the same session, that the short space of ten minutes was denied to men who had spent much time in elaborating reports, a whole hour was devoted to a display of urinary calculi by Dr. Peter Parker, and observations concerning the different grades of Chinese treated by him when in the Celestial Empire.

Perhaps such a mode of doing business is all right, and calculated to promote the interests of the association; but if so, our sense of propriety is far from being correct.

It will aid the treasury of the association, however, by diminishing the amount of matter for the forthcoming volume of transactions. The recent meeting has satisfied us more fully than before, that the plan for conducting the business of the association which we proposed three years since, and which was reported against by the chairman of the committee of arrangements, Dr. Z. Pitcher, at the opening of the meeting in Detroit, would greatly increase the interest of the annual meetings and the usefulness of the association.

